# Adapting patient treatment of neurological diseases during the COVID-19 pandemic

**DOI:** 10.3934/Neuroscience.2023006

**Published:** 2023-04-21

**Authors:** Scott Mendoza

**Affiliations:** Department of Clinical Pathology, Namseoul University, Cheonan 31020, South Korea

**Keywords:** clinical neuroscience, neurologist, neurology, SARS-CoV-2

## Abstract

Treating neurological patients during the pandemic period has become extremely challenging. At the same time, responding properly to these challenges has been diverse around the world, with varying levels of readiness, discipline, and approach. Additionally, there are significant differences in healthcare resources and processes between nations, and even within a nation, and these have significantly influenced the treatment procedure throughout the pandemic. However, neurologists have been called to care for patients with neurological symptoms who have COVID-19, and to continue managing COVID-19-affected neurological comorbidities in patients as before. This study highlights how the treatment procedures for neurological diseases are rapidly changing due to the spread of the SARS-CoV-2 virus. It also focuses on the challenges healthcare professionals are facing while providing proper treatment to neurological patients during the pandemic situation. Lastly, it offers some useful recommendations regarding the effective management of neurological diseases during the COVID-19 pandemic period.

## 1. Introduction

Epidemics have caused social and demographic changes throughout history. The bubonic plague changed the course of the Middle Ages by slowing the rate of societal advancement in addition to killing a great number of people. In a short amount of time, the public's perception of the new coronavirus, SARS-CoV-2, has shifted. Although social isolation is a vital goal, its effects have caused people to make judgments that they would never have before. Healthcare systems have been compelled to adapt quickly to the new reality, adopting choices that are in the patient's best interests but also have unintended side effects. Because of the allocation of resources to this urgent issue that is seen as decisive, care for patients with other diseases has been reduced as a result of giving patients with SARS-CoV-2 infection priority [1].

Consequently, many patients may experience unfavorable effects from the distraction effect, hence attempts are made to avoid this. According to case reports and particular investigations of hospitalized patients, the prevalence of neurological symptoms among COVID-19 patients appears to be modest. However, there is a chance that the virus may enter the nervous system by circulation, an upper transcribrial, or a conjunctival channel [2]. As neurological diseases are the primary cause of disability worldwide, the epidemic could have adverse effects on other neurological patients if they do not receive specialized care [1].

## 2. Influence of COVID-19 pandemic and confinement on neurological patients

Orsini et al. [2] highlighted that the neurological effects of SARS-CoV-2 are similar to those of other coronaviruses. The virus itself or the systemic effects of the infection may directly cause such involvement, which has been seen in more severe cases. COVID-19 could emerge with fewer usual symptoms (namely flu-like symptoms) in the elderly and in those with cardiac or endocrinological comorbidities, as opposed to more severe neurological manifestations. 78 out of 214 patients (36.4 percent) in the extensive investigation by Mao et al. [3] did exhibit neurological signs due to COVID-19. The most frequent of these symptoms were headache and dizziness, while peripheral nervous system (PNS) manifestations mostly involved changes in taste and smell (up to 5.6 and 5.1 percent of patients, respectively). Similarly, according to Nordvig et al. [4], potential neurological complications of COVID-19 include headache, fatigue, anosmia, dizziness, anorexia, ageusia, meningoencephalitis, myalgias, altered consciousness, hemorrhage, Guillain-Barré Syndrome, seizure, syncope, and stroke.

Neuromuscular disease (NMD) patients are also thought to be more vulnerable to COVID-19 problems than the general population [2]. Some of them, such as individuals who have swallowing and breathing muscles involved, have other comorbid conditions, or are receiving IST (e.g., steroids), may be more susceptible to infection. In addition, infections, like those that cause spinal muscular atrophy or myasthenia gravis, can cause diseases like NMD to worsen or develop, and those who have both NMD and acute respiratory illnesses have a considerably higher risk of respiratory failure and a greater need for intensive care [2]. Avula et al. [5], Markus and Brainin [6], Paterson et al., [7] also found that SARS-CoV-2, like other viruses and bacteria, has been linked to a number of cases of acute cerebrovascular disorders (CVD), including ischemic or hemorrhagic stroke and cerebral venous sinus thrombosis.

505 adult patients with neurological symptoms were assessed at the Emergency Department (ED) between 20 February and 30 April 2020 by Pilotto et al. [8]. It was discovered that neurological patients with COVID-19 had a poor prognosis, as evidenced by greater hospitalization rates, a high mortality rate, and higher impairment after discharge. This emphasizes the negative effects of COVID-19 on neurological patients, who are more prone to early deterioration and death regardless of age or premorbid condition due to their fragility and vulnerability. Additionally, COVID-19 infection has been specifically shown by Pilotto et al. [8] to alter the clinical presentations and treatment of neurological patients. The organization of healthcare and daily practices are both significantly impacted by this.

According to Matas-Guiu et al. [1], medical care delivery has been influenced by confinement, which was a component of the plan to stop the spread of COVID-19. This is especially important for neurological patients since they frequently have risk factors, like advanced age and disability, that raise the likelihood of a worse outcome in the event of infection. Additionally, these patients typically have a companion when they attend consultations; however, because of the pandemic, this has become extremely challenging, impacting the conventional forms of neurological treatment employed in consultations. Implementing telemedicine in patient care is difficult despite the availability of the necessary resources, even though it has become essential for healthcare team management and maintaining the delivery of neurological care while preventing the transmission of SARS-CoV-2 between patients, their relatives, nurses, neurologists, and researchers.

Without a doubt, the COVID-19 epidemic has changed how neurology is practiced and how patients with movement disorders are managed around the world. Many neurologists were forced to practice from their homes and devise new methods for managing neurological patients remotely as a result of the global lockdown. Some neurologists were forced to perform internal medicine when more staff was required. All of these improvements, though, have been incredibly difficult to implement, particularly given the need for them to be done safely, swiftly, and effectively [9]. For these reasons, clinicians are trying to make judicious decisions in deciding if the advantages of admission or intervention outweigh the use of resources and potential risks under the current crisis. The neurological intensive care units (ICUs) are often called upon for co-management, even though these beds and staff are also required for COVID-19 overﬂow. In addition, resources like thrombectomy are specifically reserved for patients that need the most. Neurologic patients with COVID-19 are also isolated from neurology patients without the disease [10]. Even in some cases, as in Italy, the majority of hospitals have reallocated neurologists for helping patients with COVID-19 [11].

## 3. New measures taken to treat patients with neurological diseases during pandemic

As found by Matas-Guiu et al. [1], various steps are taken to treat neuro patients during the pandemic period, such as creating a functional reorganization plan, using telephone consultations to maintain neurological care, making decisions about periodic in-hospital treatments and complementary testing, providing care at a unit outside the hospital for emergency patients, and prioritizing patients with epilepsy using specific telephone services. Additionally, when a large number of people are infected with the virus, it is very difficult to organize neurological emergencies and admissions. So, all concerned need to be sincere and agile in treating emergency neurological patients to enhance the effectiveness of their treatment process.

According to Venketasubramanian et al. [12] protected channels are being turned on for all patients who have infections, whether they are suspected or proven. Even if protected pathways for stroke patients with suspected or confirmed COVID-19 are set up, a lack of qualified staff may make it difficult to evaluate and keep track of these patients' neurologic condition. In this circumstance, inpatient telemedicine has emerged as a key technique for enabling stroke team members to carry out crucial neurological monitoring of patients in designated COVID-19 areas, while minimizing person-to-person contact and preserving limited PPE supplies. Using telemedicine technology, evaluations of stroke patients are carried out fully digitally both in the emergency room and during routine hospital rounds. Additionally, a telestroke network enables patients with low ABCD_2_ scores to receive treatment in outlying hospitals rather than being needlessly sent to comprehensive stroke centers, particularly when the Emergency Rooms (ERs) are overburdened with COVID-19 management tasks.

In addition to getting standard-of-care imaging, all confirmed or suspected COVID-19 patients with acute stroke are also being assessed for intravenous thrombolysis and/or intra-arterial mechanical thrombectomy (MT). All patients are needed to wear surgical masks while being transported to imaging suites, but those who have suspected COVID-19 in particular must have supplemental oxygen applied underneath the mask using nasal prongs or other similar equipment. All medical staff members who come into direct physical contact with patients must also put on the proper PPE, such as a full-sleeved gown, surgical mask, eye protection, and gloves. Additionally, the majority of medical facilities are continuing to use a uniform strategy for imaging patients who have suffered an ischemic stroke in the context of COVID-19. Facilities that previously relied on CT/CTA/CTP continue to do so, and centers that previously relied on MRI/MRA/MRP are doing the same. A COVID-19 imaging suite that can handle all patients with COVID-19 has started to be adopted by facilities with numerous CT or MRI scanners. To prevent the infection of healthy patients in CT scanners, this is the preferred way [12].

## 4. The use of technology to treat neuro patients during pandemic

It is important to analyze whether telemedicine can be used over extended periods of time to serve huge numbers of patients, as it has previously been suggested as a reaction in the event of a disaster [1]. Despite the existence of telestroke programs before the pandemic, the pandemic's situation necessitated adapting patients' access to neurological care to protect the staff caring for these patients. It was also necessary to make decisions regarding patients with stroke who also present SARS-CoV-2 infection. The usage of previously sporadic resources, including email and other electronic tools, has increased due to the need to respond to emergencies, monitor patients with chronic but changing diseases, and continue to provide therapy. The drawback of these technologies is that they are frequently not used by patients because of their advanced age or the features of their illnesses [13]. Neurologists may be able to evaluate clinical changes remotely with the help of images or videos that patients or their family members have captured; for instance, the video may be used to assess gait, balance, and oculomotor abnormalities. All of these techniques have made it possible for neurology departments to continue offering care and patient follow-up despite the situation [1].

Moro & Fernandez, [9] also contended that the clinical conditions of many patients with Parkinson's disease (PD), Huntington's disease, dystonia, or tics often deteriorate as they are unable to visit their neurologists regularly due to lockdown. To solve these problems, several actions have been taken, and telemedicine is deemed the most successful one among them. Particularly, when the treatment procedure is not time-sensitive, then telemedicine procedure is widely used due to its efficacy and ease. Moro & Fernandez, [9] described how an innovative two-tiered telemedicine model is implemented in Italy, where allied health professionals first conducted a telephone triage to determine the needs of their parkinsonian patients. This reduced the volume of patients who required more involved virtual visits with neurologists.

As argued by Chen & Hemmen [10], when treating high-risk groups, telemedicine platforms are essential. Pre-pandemic literature claimed that, for all neurology subspecialties, telemedicine was not less effective than in-person clinic visits. In response to the pandemic, Medicare coverage was extended outside rural regions, and tele-HIPAA regulations were relaxed. This sped up widespread adoption. The technology is adaptable and has been expanded to include neuro-rehabilitation, giving a hotline to prevent isolation among the elderly and disabled, and monitoring using remote equipment (such as accelerometers for Parkinson's disease). Telemedicine proponents emphasize the “4 Cs”: improved patient comfort, convenience, better access to care, and confidentiality [10].

Markus and Brainin [6] also argued that telemedicine can be effectively used for acute assessment of thrombolysis. This is an essential component of stroke therapy, especially in remote areas, and expanded telemedicine use may be helpful during the current epidemic. With this approach, the stroke team's exposure to risk is decreased, a reasonable stroke evaluation is possible, unnecessary inter-facility transfers are avoided, and the usage of mandatory personal protective equipment is avoided. Telemedicine is a good option for stroke outpatient follow-up as well as acute stroke. It permits follow-up while preserving the social isolation imposed in many nations. This can be done over the phone, but visual teleconferencing allows for better patient engagement and more limited examination. With outstanding patient experience comparable to in-person sessions, video teleconferencing has been proven to be well-suited for outpatient follow-up of patients with uncommon stroke disorders [6].

## 5. Challenges posed by COVID-19 regarding neurological treatment and research

Worldwide, neurological disorders are the main factor in disability. However, any epidemic or pandemic, like the COVID-19 one, can have detrimental effects on people who have neurological conditions. Due to the epidemic's heavy toll, these patients struggle to receive the right care. The policy of confinement has affected the delivery of medical treatment since it aims to stop the spread of pandemic diseases. The neurological community should take note of this in particular. Many patients have risk factors such as advanced age, heart conditions, diabetes mellitus, and other disability-causing conditions. These elements raise the possibility of neurological illnesses having a bad prognosis. Additionally, these patients typically need to bring companions with them for consultations, and because of the epidemic, this has become extremely challenging, impacting the conventional forms of neurological care employed in consultations [13].

Current major clinical studies related to neurology have also faced numerous difficulties as a result of COVID-19. Enrollment has been halted due to quarantine and travel restrictions, and tight study guidelines put a lot of logistical pressure on research staff. However, there is still a moral requirement to finish these research studies out of respect for the current study participants. The study group handles this in a complicated and unique way. As the pandemic passes, it will be necessary to account for and further investigate both the direct (such as the loss of participants or data) and indirect (such as the infection as a confounder) effects of the pandemic.

## 6. Recommendations

If neurologists identify that one of their patients is affected by the SARS-CoV-2 virus, then they need to be particularly careful about their treatment process, as the prognosis may be influenced by the underlying neurological condition. If new progression or symptoms of neurological disease emerge, neurologists should make the proper decision and analyze the risks and benefits of visiting the hospital, and it needs to be assessed individually. If remote follow-up can be possible and effective, then it should be prioritized by the neurologists. However, when in-hospital treatments are preferred, then there should not be any delays. In short, neurologists should analyze the case of the patients individually, consider the need for emergency in-hospital treatments, and take the appropriate action for the betterment of the patients [13]. In hospitals, a ‘secure’ off-site care unit should be created for treating patients with neurological emergencies or when continued administration of treatments becomes essential. This secure unit may also be useful when telephone consultation is not enough, like when multiple sclerosis relapses or myasthenia gravis symptoms exacerbate [1].

It is also crucial to consider the need for physical separation between the doctor and the patient during the pandemic period. Furthermore, patients should attend consultations alone whenever possible, lumbar punctures should only be performed when necessary, eye fundus examinations should be avoided whenever possible to reduce the amount of time that the doctor and patient are in close proximity, and the clinical examination should focus on the symptoms that motivated the consultation in order to keep the time spent in that proximity to a minimum. Despite these factors, certain individuals necessitate complicated therapies or crucial diagnostic procedures, requiring visits to the hospital. New, individualized forms of consultation are required in these circumstances. Prior to the consultation, patients' temperatures should be taken. Those who have a fever should be seen alone, and all safety precautions should be followed. The choice entails comparing the possibility of virus transmission against the potential advantages of the planned procedure or investigation. Patients must be followed up and given immediate access to the neurology department under certain conditions. For instance, epileptic episodes pose a problem for individuals who are isolated. In order to maximize therapy outside of the hospital, care must be improved in the case of an emergency or prospective emergency in addition to ongoing seizure monitoring and treatment. Typically, patients are provided with emergency action plans and rescue drugs, which may be administered by their families [1].

Bhaskar et al. [14] advised that following procedures need to be followed for patients with neurological symptoms during the pandemic period:

- Patients should be watched for any short-term or long-term neurological or cognitive problems. Clinicians could evaluate cognitive impairment using standard measures like the Mini-Mental State Examination (MMSE). If the test has a good sensitivity/specificity balance (>85%), it can also be administered on a large scale by telephone, informant proxy, or directly by mail [such as the Cognitive Assessment Screening Test (CAST)].
- If a patient later develops neurological symptoms, it is important to determine whether they have ever had COVID-19 infection as well as the severity of their clinical condition and any supporting imaging results.
- Additionally, imaging could be utilized to evaluate the blood-brain barrier (BBB's) damage and determine whether COVID-19 causes a temporary or permanent change. Evaluation and permeability quantification of BBB could be carried out in one of two ways: (a) semi-quantitatively by comparing scans taken before and after contrast injection; or (b) quantitatively by using perfusion-weighted or permeability magnetic resonance imaging (MRI) technique in comparison to dynamic contrast-enhanced MRI (DCE-MRI).

In order to treat patients with acute neurological conditions, Bhaskar et al. [14] also recommended using telemedicine whenever possible, maintaining social isolation of patients visiting hospitals and other facilities within clinics, and separating individuals with fever and respiratory symptoms from those without these symptoms as a way to lessen the impact. Proper training, including simulation training, should be offered to healthcare professionals regarding the treatment of neurological patients. These healthcare workers should also be given regular breaks so that they can avoid stress and fear. They should also be provided with the required information related to relaxation and coping strategies. Online peer-support communities for discussion, together with social media and messaging chat groups, may offer a useful outlet for neurologists.

Venketasubramanian et al. [12] with the involvement of a large international panel of stroke experts, evaluated the rapidly expanding body of research on COVID-19 stroke, and offered recommendations for stroke management in this difficult new environment. The recommendations included changes for prehospital emergency rescue and hyperacute care, inpatient stroke or intensive units, post-hospitalization rehabilitation, follow-up with at-risk families and communities, and multispecialty departmental developments in the allied professions.

In the context of Coronavirus disease 2019 (COVID-19), it is also crucial to address acute neurological diseases, especially acute ischemic stroke, given the risk of infection for patients and healthcare professionals as well as mounting evidence of the virus's propensity to become neuroinvasive. The safety and well-being of the patients as well as the frontline healthcare staff must be taken into account while developing procedures for the management of continuing acute neurological care and intervention. In order to reduce viral exposure to healthcare personnel and patients, Bhaskar et al. [14] looked at existing pathways and their effectiveness. They then developed a holistic method to handle patients with acute neurological disorders in the COVID-19 scenario. For instance, as suggested by Bhaskar et al. [14], when treating individuals with acute ischemic stroke during COVID-19, a conservative approach comprising fever screening, history taking to rule out COVID-19 hazards, and the presence of infectious symptoms may become essential. To reduce the danger of infection, it has been recommended to limit the number of healthcare personnel in the patient's room, set rules for wearing personal protective equipment, and assign certain duties to different people.

Neurologists interact with the COVID-19 population in addition to stroke patients for symptoms including anosmia, headache, encephalopathy, or meningitis-encephalitis. To categorize a COVID-19 patient and avoid replacing a clinical evaluation with proxy diagnostics due to the danger of infection, specific measures must be done. Ancillary testing (such as CT scans and EEG) involves employees at risk for viral exposure in addition to the equipment that needs to be sanitized. However, if necessary, the standard of care shouldn't be withheld because of COVID-19. Given the variation in risk tolerance across people, a standardized methodology may aid in removing these potential biases in the diagnosis of COVID-19 patients. Finally, because mechanical ventilators and ICU resources are required, medical teams must be realistic but thorough when predicting the prognosis of catastrophic neurologic disease to help with resource allocation [10].

Orsini et al. [2] reported that COVID-19 is linked to neurological symptoms such as vertigo, headache, taste and smell disturbances, and issues affecting the nervous system that are ultimately brought on by the pathologic processes induced by SARS-CoV-2. Younger people appear to be less likely to develop severe types of COVID-19. However, neurological symptoms have also been found in pediatric patients, and the infection sometimes had long-term neurological effects. Additionally, during this pandemic, children who are being treated with specific medications (such as immune-suppressant therapy) or who have specific neurological illnesses must be closely watched. The primary drug-drug interactions as well as the neurological adverse effects of COVID-19 therapies should be understood by neurologists.

Von Oertzen et al. [15] presented the following consensus statements from the European Academy of Neurology (EAN) experts for guiding neurologists caring for patients with COVID-19:

- Special precautions to prevent potential exposure to or contamination with SARS-CoV-2 should be taken without postponing treatment while treating acute stroke patients with endovascular therapy.
- The patient or caregiver should get in touch with the homecare/palliative team/caring ALS center and notify the doctor who regularly treats the patient in the event that respiratory decompensation occurs in patients with neuromuscular disorders, such as amyotrophic lateral sclerosis (ALS), who are on home ventilatory support or with initial respiratory symptoms.
- For a protracted duration of isolation, a sufficient supply of medication and ventilatory support equipment must be guaranteed.
- When conducting neurophysiology tests, technicians must follow all regulations set forth by the intensive care unit (ICU) medical staff, including those pertaining to droplet and airborne precautions.
- If electroencephalographic (EEG) and electromyographic (EMG) investigations are required, special sanitary settings (in accordance with contamination prevention guidelines) must be set up.
- In order to identify neurological signs and problems, neurologists must be involved in the care of COVID-19 patients from the beginning and in the intensive care unit.
- When a teleconsultation option is available, specialist consultations should be conducted over the phone. This may make it easier to determine which people require in-person appointments.
- Since the effects of respiratory infections are anticipated to be more severe than in the general population, patients with neuromuscular illnesses that specifically affect respiratory function (e.g., ALS) should be quarantined in their homes to prevent contracting the infection.
- Departments are urged to delay any elective EEG and EMG examinations unless they are urgent or likely to significantly alter management, given the lack of personal protective equipment and the potential risk of exposing patients and healthcare workers to infection. However, these choices should be handled in accordance with regional laws and regulations.
- Detergents or products containing alcohol should be used to disinfect wheelchairs, walking aids, and other surfaces. The location where clothing from the outside is collected at the entrance should be included in this as well.
- If necessary, a principal carer should be named; this person will be in charge of coordinating the patient's care. The caregiver should stay beside the patient when they are isolated from others.
- If applicable, a backup carer should be identified for each patient, limiting external contacts to reduce the risk of infection.

Several neurological conditions are directly and indirectly affected by COVID-19. To provide a clear and concise summary of these conditions and their relationship with the pandemic, two tables have been organized. Table 1 presents neurological conditions directly affected by COVID-19, detailing the references and the specific impacts of the virus on each condition. Table 2, on the other hand, lists neurological conditions indirectly affected by COVID-19, providing relevant references and explaining how the pandemic has influenced the management and outcomes of these conditions.

**Table 1.** Neurological conditions affected by COVID-19 directly.

**Condition**	**Reference**	**How COVID-19 Affects the Condition**
Guillain-Barre Syndrome	[3],[4]	Potentially triggered by COVID-19 infection
Encephalitis	[3],[10]	SARS-CoV-2 virus can cause encephalitis
Acute Necrotizing Encephalopathy	[4]	Rare complications of COVID-19 infection
Acute Ischemic Stroke	[7],[14]	Increased risk due to hypercoagulable state and endothelial dysfunction
Cerebral Venous Thrombosis	[7],[11]	Increased risk due to hypercoagulable state
Seizures	[4],[8]	Potential association with COVID-19 infection
Myasthenia Gravis	[1],[2]	Potential worsening of symptoms due to infection

**Table 2.** Neurological conditions indirectly affected by COVID-19.

**Condition**	**Reference**	**How COVID-19 Affects the Condition**
Multiple Sclerosis	[1],[15]	Disruption of routine care and management, potential exacerbation of symptoms
Parkinson's Disease	[9],[10]	Disruption of routine care, increased vulnerability to complications from COVID-19
Epilepsy	[1],[10]	Disruption of routine care, challenges in emergency management
Alzheimer's Disease	[9]	Disruption of routine care, increased vulnerability to complications from COVID-19, potential cognitive decline

## 7. Treatment of neurological symptoms resulting from long-Covid

Accumulating evidence indicates a high prevalence of prolonged neurological symptoms among COVID-19 survivors [16]–[19]. Currently, there are no standardized criteria for diagnosing “long COVID” [16],[9]. However, long COVID is viewed as a multi-organ disorder with a wide spectrum of clinical manifestations that may indicate underlying cardiovascular, pulmonary, endocrine, hematological, renal, gastrointestinal, dermatological, immunological, psychiatric, or neurological diseases.

Involvement of the central or peripheral nervous system is noted in over one-third of patients who had severe acute respiratory syndrome coronavirus 2 (SARS-CoV-2) infection, with neurological symptoms approximately three times more frequent in observational studies [16],[17]. Fatigue, cognitive impairment, headache, sleep, mood, smell or taste disorders, myalgia, sensorimotor deficits, and dysautonomia are the most frequent neurological manifestations of long COVID [16]–[19]. The pathophysiology underlying long COVID is still unclear, but neuroinflammatory and oxidative stress processes are thought to play a role in propagating long-COVID neurological sequelae [16],[18],[19]. Studies have shown that patients with long COVID may demonstrate brain hypometabolism, hypoperfusion of the cerebral cortex, and changes in the brain structure and functional connectivity [18]. Diagnostic and therapeutic algorithms may aid in the prompt recognition and management of underlying causes of neurological symptoms that persist beyond the resolution of acute COVID-19 [16],[19]. Collaborative research initiatives are urgently needed to expedite the development of preventive and therapeutic strategies for neurological long COVID sequelae, as causal treatments are currently unavailable [16],[19]. Therapeutic approaches are available for symptom-oriented management of neurological long COVID symptoms [16],[18].

Early detection and prevention of long-covid neurological sequelae are crucial for the proper long-term management of patients with preexisting neurological diseases. Further research is necessary to elucidate the pathogenesis of long COVID and to establish standardized criteria for its diagnosis [16],[19]. The high prevalence of prolonged neurological symptoms among COVID-19 survivors warrants close attention as it may drastically change the traditional treatment of patients with neurological diseases.

## 8. Conclusions

Critical neurological care during the COVID-19 pandemic is under increasing strain as a result of continuous service reorganization and rationing to address the needs of COVID-19 sufferers. In this article, a number of ideas have been put forth that could reduce the danger to healthcare systems, staff members, and patients with neurological conditions. In addition, public health initiatives need to be taken to inform and raise community awareness of the need for immediate medical assistance in the event of acute neurological symptoms. The pandemic's effects on hospitals necessitate a functional reorganization of all the concerned departments, the formulation of new goals, and the reallocation of resources and responsibilities. Consequently, department employees must be included in this process to ensure they are aware of and accept specific roles. Careful steps need to be taken by the doctors, nurses, and all concerned so that appropriate treatment can be offered to neurological patients during this critical period of time. As more time passes, new challenges stemming from long COVID will need to be understood and treatment of neurological patients adapted accordingly.

In conclusion, it can be said that the COVID-19 storm has presented enormous challenges for neurologists and their patients everywhere in the world. The destruction brought on by the epidemic may have varied greatly depending on the starting points and healthcare systems in each place, but finally, the strength of data sharing, inventiveness, and resiliency won out. “Adaptive neurology” has become the most potent weapon against this invisible foe.
